# Immuno-imaging of ICAM-1 in tumours by SPECT

**DOI:** 10.1016/j.nucmedbio.2020.02.014

**Published:** 2020

**Authors:** Michael Mosley, Julia Baguña Torres, Danny Allen, Bart Cornelissen

**Affiliations:** Oxford Institute for Radiation Oncology, Department of Oncology, University of Oxford, Old Road Campus Research Building, Roosevelt Drive, Oxford OX3 7DQ, United Kingdom of Great Britain and Northern Ireland

**Keywords:** ICAM-1, Pancreatic cancer, SPECT imaging

## Abstract

**Purpose:**

Molecular imaging of cancer cells' reaction to radiation damage can provide a non-invasive measure of tumour response to treatment. The cell surface glycoprotein ICAM-1 (CD54) was identified as a potential radiation response marker. SPECT imaging using an ^111^In-radiolabelled anti-ICAM-1 antibody was explored.

**Methods:**

PSN-1 cells were irradiated (10 Gy), and protein expression changes were investigated using an antibody array on cell lysates 24 h later. Results were confirmed by western blot, flow cytometry and immunofluorescence. We confirmed the affinity of an ^111^In-labelled anti-ICAM-1 antibody *in vitro*, and *in vivo*, in PSN-1-xenograft bearing mice. The xenografts were irradiated (0 or 10 Gy), and [^111^In]In-anti-ICAM-1 SPECT/CT images were acquired 24, 48 and 72 h after intravenous administration.

**Results:**

ICAM-1 was identified as a potential marker of radiation treatment using an antibody array in PSN-1 cell lysates following irradiation, showing a significant increase in ICAM-1 signal compared to non-irradiated cells. Western blot and immunohistochemistry confirmed this upregulation, with an up to 20-fold increase in ICAM-1 signal. Radiolabelled anti-ICAM-1 bound to ICAM-1 expressing cells with good affinity (K_d_ = 24.0 ± 4.0 nM). [^111^In]In-anti-ICAM-1 uptake in tumours at 72 h post injection was approximately 3-fold higher than non-specific isotype-matched [^111^In]In-mIgG2a control (19.3 ± 2.5%ID/g *versus* 6.3 ± 2.2%ID/g, P = 0.0002). However, ICAM1 levels, and [^111^In]In-anti-ICAM-1 uptake in tumours was no different after irradiation (uptake 9.2%ID/g *versus* 14.8%ID/g). Western blots of the xenograft lysates showed no significant differences, confirming these results.

**Conclusion:**

Imaging of ICAM-1 is feasible in mouse models of pancreatic cancer. Although ICAM-1 is upregulated post-irradiation in *in vitro* models of pancreatic cancer, it shows little change in expression in an *in vivo* mouse xenograft model.

## 1 Introduction

Radiotherapy (RT) using ionizing radiation (IR) is an essential component of treatment for more than half of all newly diagnosed cancer patients, in whom it can irreversibly damage targeted tumour cell DNA to reduce tumour size [1]. Real-time molecular imaging of cellular markers of IR damage would provide a non-invasive measure of tumourigenesis, RT sensitivity and tumour status in response to treatment, and would be useful in tailoring RT based on tumour biology in individual patients [2]. Tumour response to treatment varies between patients and anatomical changes may not be measurable using anatomical imaging techniques such as CT or MRI, for a significant period following treatment [3]. This will impact on the timely need to consider changes to treatment in order to improve patient outcome, should the treatment prove non-efficacious. Thus far, there exist only a limited number of molecular biomarkers that guide clinical decisions following radiation therapy [4,5], especially those imaging extracellular epitopes to facilitate imaging. This therefore highlights the need for alternative imaging biomarkers.

Here, we employed a human pancreatic cancer model, using the pancreatic ductal adenocarcinoma (PDAC) PSN-1 cell line to identify cell-surface markers of radiation damage, in an *in vitro* screen. Given that there is an urgent clinical need to improve survival rates for pancreatic cancer patients since these are particularly low in PDAC (a 5-year survival rate of <5% [6]) - in large part because of late diagnosis and the therapy resistance of the disease [7]. Fast decision-making regarding the efficacy of any therapy, including radiotherapy, is therefore paramount in order to adapt or adjust treatment in a timely fashion [8,9]. PSN-1 cells are not particularly radiosensitive or radioresistant [10].

We report our results after the screening of an antibody array comparing PSN-1 cell lysates obtained before and after irradiation. This identified ICAM-1 (Intercellular Adhesion Molecule 1) as being up-regulated in irradiated cells. ICAM-1 is a cell surface glycoprotein, a member of the immunoglobulin superfamily, and is typically expressed on endothelial cells and cells of the immune system [11]. In addition, it is reported to be involved in tumour metastasis, cancer progression and is an independent prognostic factor [12]. Expression of ICAM-1 is further increased in metastases of the liver [13], where it mediates the formation of a pro-metastatic niche by endothelial cell activation of signalling pathways [14], assisting tumour cell extravasation [15,16], and the recruitment of immune cell populations [17]. Additionally, ICAM-1 is overexpressed in triple-negative breast cancer tissue and was previously imaged by near-infrared fluorescence in a mouse xenograft model [18]. Finally, ICAM-1 expression had previously been shown to be upregulated after gamma irradiation of human multiple myeloma cells, potentially leading to an increase in the immunogenicity of tumour cells [19].

This report describes the identification of proteins whose expression on pancreatic cancer cell-lines was increased after gamma irradiation, and the selection of one of these molecules, ICAM-1, for further *in vitro* and *in vivo* characterization, and imaging using an ^111^In-labelled anti-ICAM-1 antibody.

## 2 Materials and methods

The PSN-1 pancreatic adenocarcinoma cell line was obtained from ATCC, validated by STR, and maintained at 37 °C and 5% CO_2_ in RPMI media (Life Technologies) supplemented with 10% Fetal Bovine Serum (FBS) and L-glutamate (Sigma) up to a maximum passage number of 20 following resuscitation from liquid nitrogen storage. Cells were monitored regularly for the absence of mycoplasma. Cells were irradiated using an IBL 637 Cesium-137 γ-ray irradiator to a radiation absorbed dose of 10 Gy (1 Gy/min).

### 2.1 Screening using the human oncology antibody array

PSN-1 cells in a T175 flask were irradiated (10 Gy or mock irradiated as a negative control sample; mock-treated samples were treated in an identical fashion to those that were irradiated, but without the radiation equipment turned on), and allowed to recover in a CO_2_-incubator at 37 °C for 24 h. The cells were washed in PBS, scraped and collected in a centrifuge tube and pelleted at 400 ×*g* and immediately processed or snap-frozen to −80 °C for storage. The cell pellets were lysed using standard RIPA buffer (with added COMPLETE protease inhibitors [Sigma]), centrifuged at 20,000 ×*g* to remove debris and stored at −20 °C until further use. The Proteome Profiler Human XL Oncology Antibody Array (#ARY026, Bio-Techne) allows for the measurement of 84 oncology-related proteins in a single sample. Lysates (irradiated and mock-irradiated) were incubated with the Array membranes according to the manufacturer's protocol. Briefly, 1 mg total protein was incubated with the pre-blocked antibody array overnight at 4 °C. Membranes were washed, incubated with the detection antibody cocktail, washed once more, developed and exposed to photographic film for up to 10 min. The signal intensities corresponding to the relative abundance of an antibody-antigen complex were quantified using Matlab software developed in-house [https://www.mathworks.com/matlabcentral/fileexchange/35128-protein-array-tool].

### 2.2 Western blot

Western blot was used to confirm antibody array results. PSN-1 cells were irradiated and total cell lysates prepared as described above, on three separate occasions. For this experiment cell recovery times of 2, 24, 48 and 72 h post-irradiation were used, and a separate mock-irradiated control included. Separate 8% Bis-Tris/MOPS PAGE gels (Life Technologies) were loaded with 25 μg of lysate protein per well, and blotted onto PVDF membranes (Invitrogen iBlot-2). The blots were blocked in 5% Bovine Serum Albumin (BSA) at room temperature for 1 h, and incubated with a mouse anti-human ICAM-1 monoclonal antibody (Abcam, #2213 [MEM-111]) at 1:800 dilution at 4 °C overnight. After washing the membranes with PBS, they were incubated with an anti-mouse HRP-conjugated secondary antibody (Bio-Techne, HAF007) at 1:1000 at room temperature for 1 h, and further washes followed. The blots were developed using the SuperSignal West Pico PLUS Chemiluminescent Substrate kit (#34580, Thermo Scientific), and exposed to a Li-Cor 3600 Blot Scanner*.* Similar western immunoblot analysis of ICAM-1 expression was performed using total cell lysates purified from PSN-1 xenografts.

### 2.3 Immunofluorescence microscopy

PSN-1 cells were seeded in 8-chamber slides (#IB-80841, Thistle Scientific), and 24 h later were irradiated (10 Gy), or mock-irradiated and allowed to recover for 24 or 48 h, or mock irradiated. The cells were washed and fixed in 4% formaldehyde solution (#252549, Sigma), blocked with 2% BSA for 1 h at room temperature, and stained with anti-ICAM-1 antibody 2213 (Abcam) at 1:100 dilution overnight at 4 °C, followed by Alexa Fluor 488 Goat Anti-Mouse IgG (H & L) antibody (#A11001, Life Technologies) at 1:500 dilution for 1 h at room temperature, in blocking buffer, and washes performed with PBS. Immunofluorescence microscopy was performed on a Leica SP8 confocal microscope system. The level of cellular fluorescence from the Leica fluorescence microscopy images was determined in ImageJ by calculating the corrected total cell fluorescence (CTCF) of each image.

### 2.4 Flow cytometry

PSN-1 cells were cultured in flasks to a maximum of 70% confluency, irradiated (10 Gy, or mock-irradiated) and allowed to recover for 24 h. The cells were removed from the flasks using trypsin-free Accutase dissociation solution (#A6964, Sigma-Aldrich), blocked in 1% BSA/PBS solution for 1 h at room temperature. Increasing amounts of anti-ICAM-1 antibody 2213 (Abcam) were exposed to 1 million cells in 200 μL aliquots in blocking buffer for 1 h at room temperature, followed by Alexa Fluor 488 Goat anti-Mouse antibody A11001 at 1:300 dilution in blocking buffer for 1 h at room temperature. Multiple washes in block buffer following centrifugation of the cells at 400 ×*g* were performed between treatments. Intensity histograms were acquired on a FACSCalibur Flow Cytometer (Becton Dickinson BD), and data analysed using FlowJo software (Becton Dickinson BD).

### 2.5 Radiolabelled antibody

Anti-ICAM-1 antibody (#2213, Abcam), and an isotype-matched antibody (#02-6200, Thermo Fisher Scientific), were radiolabelled with ^111^In as previously described [20]. Briefly, 0.3 mg anti-ICAM-1 antibody (#2213, Abcam) was reacted with a 20-fold molar excess of p-SCN-Bn-DTPA in chelex-treated 0.1 M sodium bicarbonate buffer pH 8.6 for 1 h at 37 °C, and the complex purified on a 1 mL sephadex G50 column, using 0.5 M MES buffer as the eluent. The DTPA-conjugated antibody was then concentrated using an Amicon Ultra 0.5 mL 30 K MWCO filter unit. DTPA-conjugated antibody (0.1 mg) was radiolabelled with ^111^In, using 0.5–1.0 MBq per microgram of antibody, for 1 h at room temperature. Radiolabelling efficiencies of >95% were confirmed by iTLC, and final antibody conjugate concentration were measured by Nanodrop spectrophotometry (Thermo Fisher Scientific).

### 2.6 *In vitro* saturation binding

PSN-1 cells were cultured in 24-well plates at 1 × 10^4^ cells/well and allowed to adhere for 24 h. Cells were then irradiated (10 Gy or mock-irradiated) and allowed to recover for 24 h, and exposed to increasing concentrations of [^111^In]In-anti-ICAM-1, or [^111^In]In-mIgG2a control in PBS for 1 h at room temperature. The cells were washed with PBS, lysed in 0.1 M NaOH and counted on a gamma-counter (Perkin Elmer 2480^2^).

### 2.7 *In vivo* SPECT imaging

All animal procedures were performed in accordance with the UK Animals (Scientific Procedures) Act 1986 and with local ethical committee approval. Xenograft tumours were established in the right hind flank of female athymic BALB/c nu/nu mice (Harlan) by subcutaneous injection of PSN-1 cells (2 × 10^6^) in a 100 μL solution of a 1:1 mixture of Matrigel (#356234, Corning) and PBS. When tumours reached a diameter of approximately 6 mm or greater, xenografts were irradiated using a Gulmay 320 kV system (2.0 Gy/min) to a radiation absorbed dose of 10 Gy. The radiation set-up allowed irradiation of the right hind quarter, including the tumour and right leg, only. Control animals were mock-irradiated.

One hour later, [^111^In]In-anti-ICAM-1 or [^111^In]In-mIgG2a (5 MBq, 5 μg) in sterile PBS (100 μL) was injected intravenously *via* the lateral tail vein (n = 3 per group). SPECT/CT images were acquired at 24, 48, and 72 h after injection, using a VECTor^4^CT scanner (MILabs, Utrecht, the Netherlands). Animals were anaesthetised by 4% isoflurane gas (0.5 L/min O_2_) and maintained at 2% and 37 °C throughout the imaging session. The temperature of the animals was maintained at 37 °C, using a custom-built mouse cradle. Image acquisition was performed over 12–17 min using a 1.8 mm pinhole rat collimator (1 frame, 100,000 counts/projection, 35–50 s/bed position). Whole-body CT images were acquired for anatomical reference and attenuation correction (55 kVp, 0.19 mA, 20 ms). Reconstruction of the SPECT images was performed using a γ-ray energy window of 156–190 keV (background weight 2.5), 0.8 mm^3^ voxels, 2 subsets, and 4 iterations using the manufacturer's SROSEM reconstruction type. To allow quantification of the SPECT data, calibration factors derived from ^111^In phantoms were used. SPECT images were each registered to CT and then attenuation corrected. Quantification of SPECT images using volume-of-interest (VOI) analyses were performed using the PMod software package (Version 3.807, PMOD Technologies), to calculate the percentage of the injected dose per millilitre per VOI (%ID/mL).

After the final imaging session, mice were euthanized by cervical dislocation, and selected organs, tissues and blood were removed. The amount of radioactivity in each organ was measured using a HiDex gamma counter (Perkin Elmer). Counts per minute were converted into MBq using a calibration curve generated from known standards and the percentage of the injected dose per gram (%ID/g) in each sample was calculated.

### 2.8 *Ex vivo* autoradiography of xenografts

After imaging, xenograft tissue from mice was flash-frozen with dry ice and stored at −80 °C overnight. Frozen tissue was sectioned (8 μm) using an OTF5000 cryotome (Bright Instruments Ltd). Tissue sections were thaw-mounted onto Superfrost PLUS glass microscope slides (Menzel-Glaser, Thermo Scientific) and allowed to dry at room temperature. The slides were then exposed to a storage phosphor screen (PerkinElmer, Super Resolution, 12.5 × 25.2 cm) in a standard X-ray cassette for 15 h, and the screen imaged using a Cyclone® Plus Storage Phosphor System (PerkinElmer). Autoradiographs were analysed using ImageJ (NIH). After autoradiography, ICAM-1 levels in *ex vivo* tissue were characterized by immunofluorescence. On a separate occasion a second cohort of 7 tumour xenografts were prepared as above that were irradiated or mock-irradiated (10 Gy). Tumour tissue of these animals was harvested at 24, 48 and 72 h post-irradiation for western blot analysis of ICAM-1, as described above.

### 2.9 Statistical analyses

All statistical analyses and nonlinear regressions were performed using GraphPad Prism v8 (GraphPad Software). One- or two-way ANOVA was used for multiple comparisons, the percentage of the injected dose per gram. F-tests were used to compare best-fit values obtained from curve-fits. All data were obtained in at least triplicate and results reported and graphed as mean ± standard deviation.

## 3 Results

### 3.1 ICAM-1 protein expression is upregulated in PSN-1 cells following irradiation

**Fig. 1:**
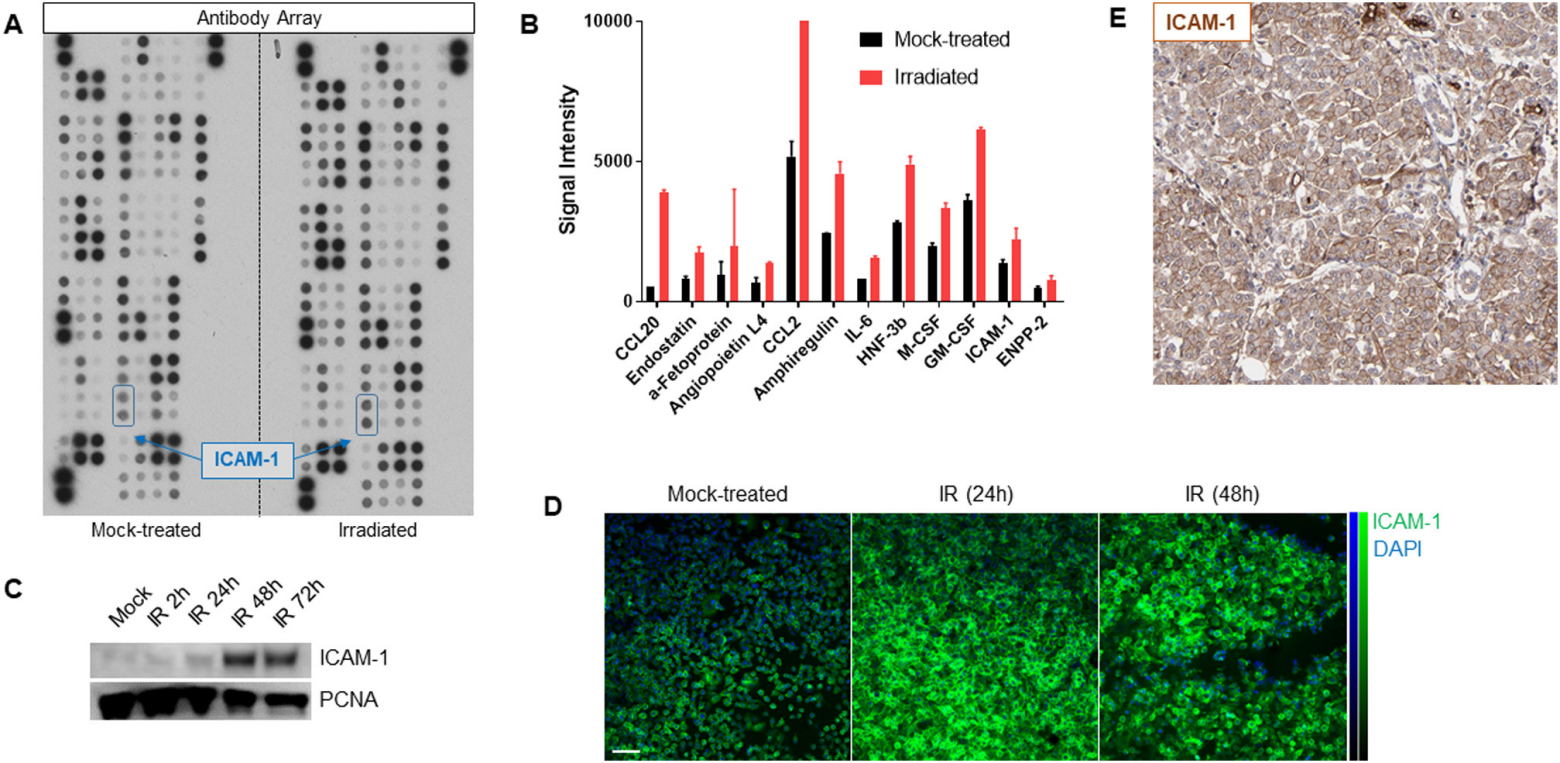
(A) Human oncology antibody array after incubation with mock-treated and irradiated lysates – ICAM-1 signals highlighted. (B) Proteins from antibody array with ≥2-fold increase in expression post-irradiation. (C) A representative western blot showing a maximum increase in signal in PSN-1 cell lysates at the 48 h time-point post-irradiation treatment (IR) when compared to the mock-treated sample. (D) Immunofluorescence microscopy (20×) shows an increase in ICAM-1 signal in PSN-1 cells post-irradiation, with a maximum increase at the 24 h time-point. Scale bar 40 μm. (E) Immunohistochemistry micrograph staining for ICAM-1 in a human PDAC section.

Quantification of the antibody array signal allowed comparison of relative protein levels in irradiated (10 Gy) *versus* mock-irradiated PSN-1 cells. This identified a number of proteins that exhibited an up to 7-fold increase in expression levels in irradiated cells. Amongst these, the cell surface protein ICAM-1 showed a marked and significant increase (Fig. 1A, B). This was confirmed by western blot on an independent set of cell lysates (Fig. 1C), demonstrating a 4.7 and a 20-fold increase in ICAM-1 expression 24 or 48 h after irradiation, respectively. Analysis of cell lysates may overestimate the amount of ICAM-1 expressed at the cell surface. Further investigation by immunofluorescence microscopy – without permeabilisation of the cell membrane – corroborated this result, with a maximum increase at the 24 h point of 2.7-fold (Fig. 1D). ICAM-1 expression was previously also found in PDAC patient samples (Fig. 1E, adapted from ProteinAtlas.org).

(Adapted from ProteinAtlas.org).

### 3.2 Anti-ICAM-1 and [^111^In]In-anti-ICAM-1 antibody characterization

**Fig. 2:**
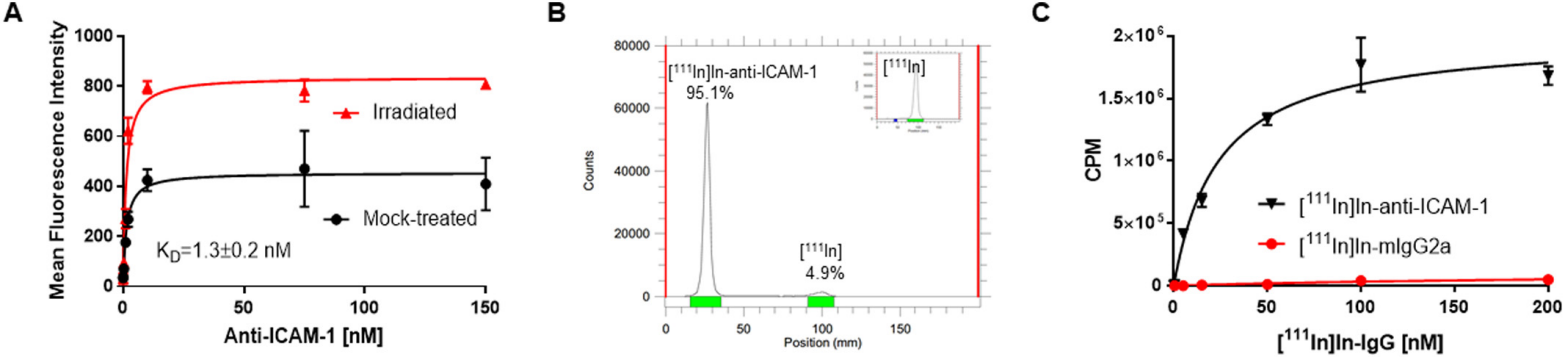
(A) Calculation of the maximum specific binding (B_max_) and affinity (K_d_) of anti-ICAM-1 antibody using flow cytometry, shows a 1.8-fold increase in B_max_ value when comparing mock-treated to irradiated PSN-1 cells. (B) iTLC of ^111^In-labelled anti-ICAM-1 antibody, showing efficient conjugation of >95%. (C) The binding affinity of the anti-ICAM-1 DTPA/^111^In radio-conjugate ([^111^In]In-anti-ICAM-1), at 24 h time-point was determined using an *in vitro* saturation binding methodology. A negative control [^111^In]In-mIgG2a was also characterized.

A commercially available anti-ICAM-1 antibody (#2213, Abcam) was selected to function as a targeting vector for a radiolabelled imaging agent. Evaluation of the antibody by flow cytometry and by saturation binding assay, showed an affinity (K_d_) of 1.38 ± 0.39 nM (Fig. 2A). Anti-ICAM-1 antibody bound significantly more to irradiated cells than non-irradiated cells (mean fluorescence intensity of 836.9 ± 32.3 *versus* 454.6 ± 27.2, respectively; P < 0.0001).

Radiolabelling with ^111^In following DTPA conjugation, resulting in [^111^In]In-anti-ICAM-1 (Fig. 2B), allowed a radioligand saturation binding assay to be performed (Fig. 2C). The affinity K_d_ of the radiolabelled antibody was calculated as 24.0 ± 4.0 nM. A radiolabelled non-selective control antibody, [^111^In]In-mIgG2a, bound significantly less to PSN-1 cells (P < 0.0001).

### 3.3 SPECT imaging shows specific ICAM-1 tumour signal but no difference between untreated and irradiated tumours

**Fig. 3:**
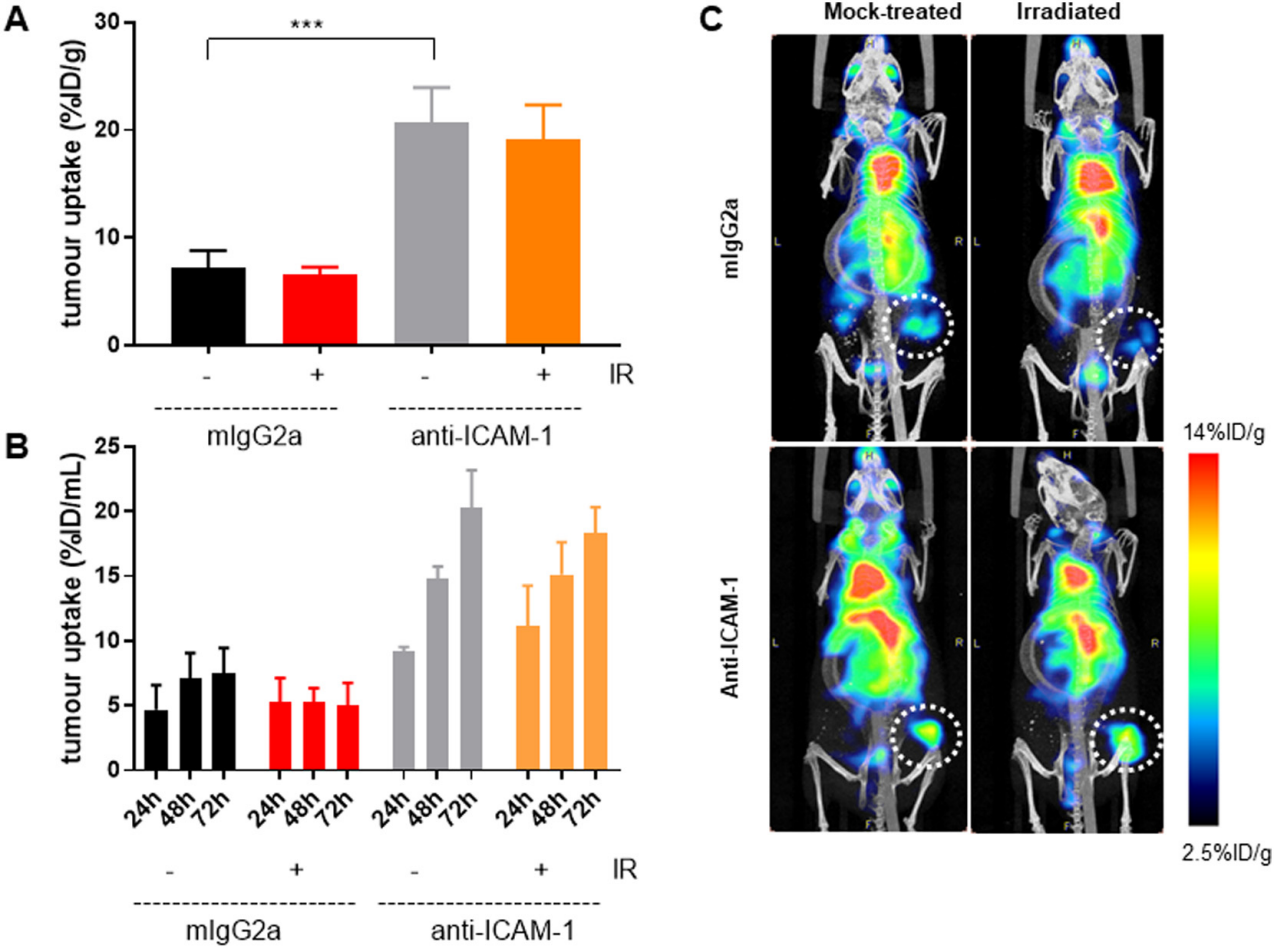
(A) Tumour biodistribution quantification shows an increase in mean percentage Injected Dose per tumour weight (%ID/g) in mice injected with [^111^In]In-anti-ICAM-1 compared to [^111^In]In-mIgG2a. However, there was no significant difference in [^111^In]In-anti-ICAM-1 uptake in the irradiated tumours compared to the mock-treated control. (B) SPECT image VOI quantification of each xenograft tumour at each SPECT time-point of 24, 48 and 72 h shows an increase in [^111^In]In-anti-ICAM-1 compared to [^111^In]mIgG2a at each of the time-points. There was a small increase in signal at the 24 h time-point in untreated mice compared to irradiated mice. However, there was no difference in [^111^In]In-anti-ICAM-1 signal in the irradiated tumours compared to the mock-treated control ones in the other 2 time-points. (C) Representative MIP images from the 24 h SPECT imaging study (white dashed circles indicate tumours).

**Fig. 4:**
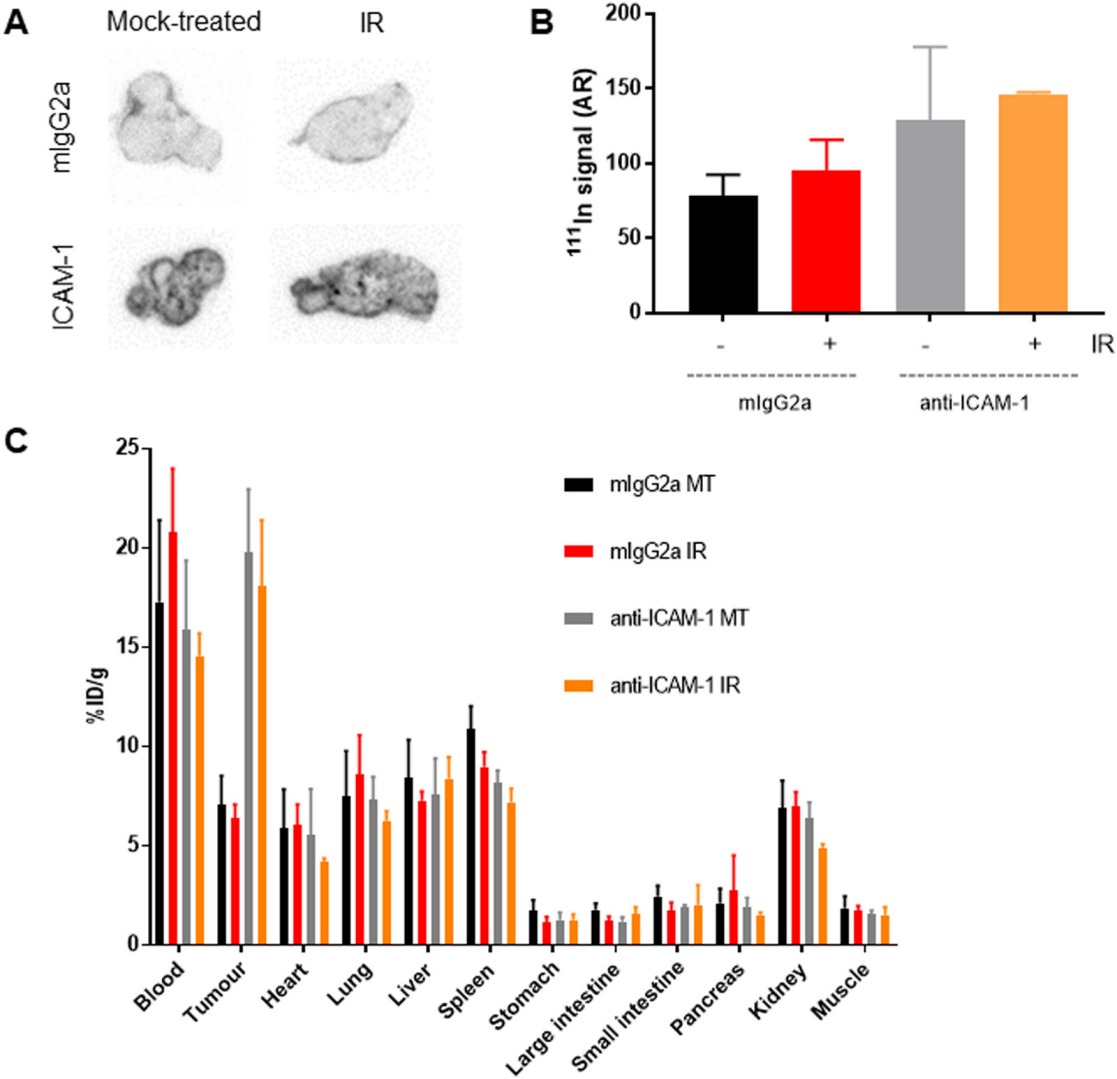
(A) Autoradiography of a representative xenograft tumour sections confirming specific uptake of [^111^In]In-anti-ICAM-1. There is a small increase in uptake of the [^111^In]anti-ICAM-1 antibody compared to the [^111^In]In-mIgG2a negative control conjugate, but little difference in uptake of [^111^In]In-anti-ICAM-1 in xenografts that had been irradiated (IR) compared to mock-treated (MT). (B) Quantification of autoradiography image in (A). (C) Biodistribution quantification (%ID/g) in mice injected with [^111^In]In-anti-ICAM-1 compared to [^111^In]In-mIgG2a, at 72 h post-injection of each IgG molecule. Tumour was either irradiated (IR) or mock treated (MT).

Quantification of the biodistribution [^111^In]In-anti-ICAM-1 and [^111^In]In-mIgG2a showed significantly higher uptake in PSN-1 tumour xenografts of the former compared to the latter, at 72 h post administration (19.9 ± 1.2 *versus* 6.9 ± 0.5%ID/g; P < 0.0001) (Fig. 3A). Conversely, and surprisingly, no significant difference was observed in [^111^In]In-anti-ICAM-1 uptake in the irradiated tumours compared to mock-treated controls (19.2 ± 1.8 *versus* 20.7 ± 1.9%ID/g, P > 0.5). Quantification of SPECT images using VOI analysis confirmed this result (Fig. 3B, C). SPECT image quantification by VOI analysis of images acquired 24, 48 and 72 h post administration confirmed these results (20.3 ± 2.9%ID/mL *versus* 18.3 ± 2.0%ID/mL in mock-irradiated *versus* irradiated mice at 72 h post injection; P > 0.05). Fig. 3C shows representative MIP images from the SPECT imaging study. Full data of the biodistribution of [^111^In]In-anti-ICAM-1 and [^111^In]In-mIgG2a is shown in Fig. 4.

Representative sections of xenograft tumours were processed for autoradiography that confirmed heterogeneous uptake of [^111^In]In-anti-ICAM-1 in PSN-1 tumours (Fig. 4A). Densitometry confirmed higher uptake of [^111^In]In-anti-ICAM-1 compared to the [^111^In]IgG2a negative control conjugate, but little difference between irradiated and non-irradiated xenografts (Fig. 4B).

**Fig. 5:**
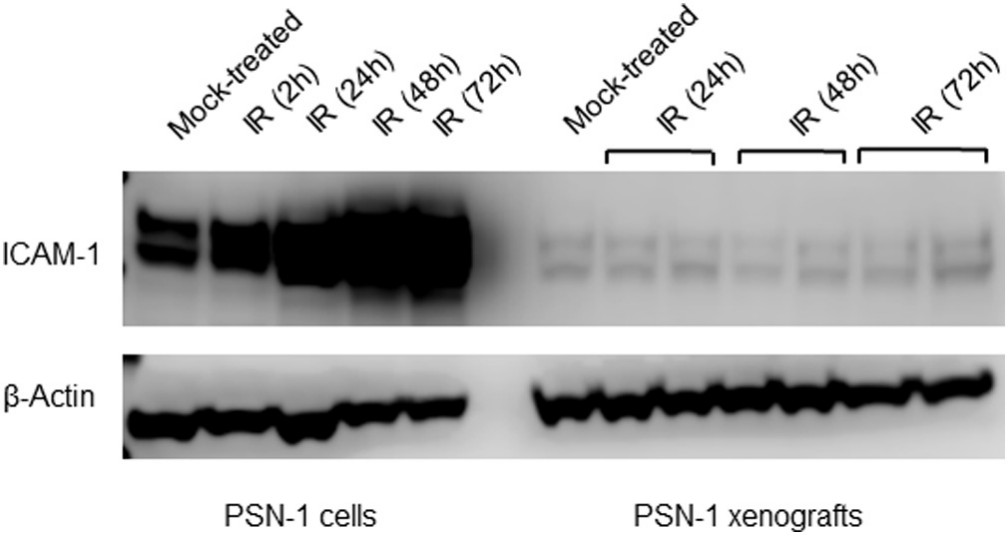
Western blot analysis of total lysates from the xenograft tumours obtained in this study, compared with *in vitro* prepared PSN-1 cells, both untreated and irradiated. The blot shows that expression of ICAM-1 was generally lower in the PSN-1 xenografts when compared to the cell cultures, and that in the xenografts there was little change in expression post-irradiation when compared to the untreated tumours.

Western blot analysis of total cell lysates from the xenograft tumours (from a second cohort) showed that ICAM-1 protein expression was far lower in the xenografts when compared to *in vitro* cell cultures, and confirmed that there was little change in this expression level post-irradiation (Fig. 5).

## 4 Discussion

This study sought to identify human tumour cell-surface biomarkers of radiation treatment (RT). Such markers would provide a non-invasive measuring tool, using immuno-PET or SPECT imaging, of tumour response to radiation treatment, as an alternative to some of the intranuclearly targeted imaging agents that bind DNA-damage associated nuclear proteins we presented before [5,21]. Pancreatic tumour cells were used as a model system because of the urgent clinical need to improve the particularly poor survival rates for pancreatic cancer patients [22,23]. Here, we provide a proof-of-principle validation of imaging of the adhesion molecule ICAM-1, using a radiolabelled antibody for SPECT imaging.

ICAM-1 is a cell surface immunoglobulin glycoprotein, typically expressed on endothelial cells and cells of the immune system [11], and its extracellular location, anchored in the cell membrane, makes it a particularly attractive target for molecular immuno-imaging [24,25]. Radiation doses ranging from 2 to 20 Gy are known to induce ICAM-1 expression in various cells *in vitro*, such as HUVECs, bone marrow ECs, and HDMECs [26]. The identification of ICAM-1 as a potential biomarker of radiation treatment is of particular interest given the involvement of adhesion molecules in the immune response following tumour RT. Irradiation can also induce expression of adhesion molecules on tumour blood and lymphatic vessels, where they play a role in leucocyte migration and extravasation into the tumour [[27], [28], [29]]. Radiation-induced ICAM-1 mediates the transmigration of tumour-promoting CD11b + myeloid cells [30]. Irradiation of a number of human cancer cells has been shown to induce ICAM-1 expression, which enhances activated NK cell-mediated cytotoxicity [31].

Here, we showed that ICAM-1 expression levels in all *in vitro* assays showed a consistent increase after 10 Gy irradiation (Fig. 1, Fig. 2) – up to a 20-fold increase in western blot analyses. We also showed proof-of-principle that [^111^In]In-anti-ICAM-1, but not the negative control [^111^In]In-mIgG2a was taken up in PSN-1 xenografts. Surprisingly, however, we only observed an increased uptake of [^111^In]In-anti-ICAM-1 in the irradiated tumour on one out of three mice in that group in our *in vivo* study. Possible explanations for this lack of radiation-induced effect may include: (1) a change in affinity for its target after conjugation and labelling of the ICAM-1 antibody, (2) an increase in ICAM-1 expression in the untreated PSN-1 cells after growth as a xenograft in inoculated mice, or (3) interference in the *in vivo* ICAM-1 signal caused by murine ICAM-1 expressed by the host (which may be altered by the presence of the xenograft tumour).

The possibility that conjugation and radio-labelling of the ICAM-1 antibody had an effect on its affinity to its target was investigated. *In vitro* saturation binding showed that [^111^In]In-anti-ICAM-1 presented a K_d_ value of 24.0 ± 4.0 nM (Fig. 2C), although presenting a reduce affinity compared to its unmodified form, still suitable for *in vivo* imaging. To determine whether the DTPA-conjugation process might explain the difference, we repeated the characterization after decreasing the molar quantity of p-SCN-Bn-DTPA used in the conjugation, but without improvement in K_d_ (data not shown). Taken together, our data suggest that the lack of radiation-induced effect is not caused by modification of the antibody for radiolabelling.

There have been previously published examples of differences in specific gene expression between *in vitro* and *in vivo* disease models. A gene expression profiling study of xenografts [32] identified changes in expression levels of numerous genes after growth in mice, in particular a marked decline in ABCB1 transporter levels in HCT-15 colon xenograft. Furthermore, differences in expression profiles of the adhesion molecules ICAM-1 and VCAM-1, in *in vitro versus in vivo* models of arteriovenous malformation disease, after treatment with ionizing irradiation, have been previously reported [26], in which already raised levels of ICAM-1 in the *in vivo* model made changes in expression levels due to irradiation treatment difficult to measure. This possibility was tested in our study, which included a cohort of irradiated xenograft bearing mice where the levels of ICAM-1 expression at various times after irradiation were measured by western blot (Fig. 5). We observed however that, although there were no significant changes in ICAM-1 expression in the irradiated xenografts compared to the mock-treated, overall ICAM-1 levels were lower in the xenograft tumour tissue compared to cell lysates from *in vitro* models (Fig. 1, Fig. 5).

Interference in the ICAM-1 signal obtained by the ICAM-1 monoclonal antibody by mouse-specific expression of the ICAM-1 molecule *in vivo* (which may be altered by the presence of the xenograft tumour) has not been excluded. However, the antibody used in this study is reported to be human-specific. Furthermore, we [25] and others [33] have previously used murine antibodies for imaging *in vivo* in mouse without non-specific background issues.

## 5 Conclusion

We report the identification of ICAM-1 as a potential biomarker of radiation treatment of the human pancreatic cancer cell-line PSN-1 *in vitro*. *In vitro* assays suggest an increase in ICAM-1 expression following 10 Gy gamma irradiation. However, although *in vivo* SPECT imaging confirms the presence of ICAM-1 in PSN-1 cells, and its suitability as a pancreatic cancer cell marker, total and radiation-mediated ICAM-1 expression is reduced *in vivo* compared to *in vitro*.

## Financial support

This research was supported by 10.13039/501100000289CRUK through the Oxford Institute for Radiation Oncology (MA, DA, GD, VK, BC). Further support was obtained from 10.13039/100011704Pancreatic Cancer Research Fund (BC, JBT).

## Declaration of competing interest

The authors disclose no potential conflicts of interest.
